# Case Report: Treating obstructive sleep apnea with maxillomandibular advancement surgery in a case with a previously reconstructed mandible

**DOI:** 10.3389/fsurg.2026.1758556

**Published:** 2026-03-25

**Authors:** Ning Zhou, Jean-Pierre T. F. Ho, Cornelis Klop, J. Peter van Maanen, Jan de Lange

**Affiliations:** 1 Department of Oral and Maxillofacial Surgery, Amsterdam University Medical Centers (UMC), University of Amsterdam, Amsterdam, Netherlands; 2 Academic Centre for Dentistry Amsterdam (ACTA), University of Amsterdam and Vrije Universiteit Amsterdam, Amsterdam, Netherlands; 3 Department of Oral and Maxillofacial Surgery, Northwest Clinics, Alkmaar, Netherlands; 4 Department of Otorhinolaryngology-Head and Neck Surgery, OLVG, Amsterdam, Netherlands

**Keywords:** maxillomandibular advancement, obstructive sleep apnea, osteotomy, reconstruction, splintless orthognathic

## Abstract

**Background:**

Maxillomandibular advancement (MMA) serves as a surgical option for the management of obstructive sleep apnea (OSA). However, its application in patients with a previously reconstructed mandible is rarely reported and technically challenging. This paper reports a case of MMA for OSA in a patient who had undergone mandibular reconstruction.

**Clinical presentation:**

A 53-year-old male, who previously received mandibular reconstruction with a fibula flap, underwent MMA for severe OSA [apnea hypopnea index (AHI) = 35.2]. Virtual surgical planning with patient-specific guides and osteosynthesis plates were used to enable precise maxillary and mandibular advancement. Post-surgery, AHI decreased to 17.6, markedly improving daytime sleepiness. Complications were minimal, limited to transient paraesthesia and osteosynthesis material removal.

**Conclusion:**

This case suggests that, when carefully planned and executed, MMA using patient-specific osteotomy guides and osteosynthesis plates can be a feasible, effective, and surgically safe treatment option for OSA in selected patients with a previously reconstructed mandible.

## 1 Introduction

Maxillomandibular advancement (MMA) surgery is a specific type of orthognathic surgery, which was originally described for the correction of dentofacial deformities and is now frequently used for the treatment of obstructive sleep apnea (OSA) (1). As the most common sleep-related breathing disorder, OSA is characterized by repetitive episodes of complete (apnea) or partial collapse (hypopnea) of the upper airway during sleep (2). The consequences of untreated and undiagnosed OSA can be serious, which include, among others, increased risk of cardiovascular disease, stroke, metabolic disease, traffic accident, and death (2).

In MMA surgery, a Le Fort I osteotomy and a bilateral sagittal split osteotomy (BSSO) are performed to advance the maxilla and mandible (1). The rationale of MMA in treating OSA is enlarging the pharyngeal space and creating more tension in the upper airway soft tissue by physically expanding the facial skeletal framework (3, 4). The current gold standard for orthognathic surgery is to virtually plan the procedure in three dimensions (3D) and transfer the surgical planning to the surgery with 3D-printed surgical splints (5). However, in patients with missing teeth, altered occlusion, or complex reconstructive anatomy, splint-based transfer may be unreliable or impractical. To overcome these limitations, splintless orthognathic techniques using patient-specific osteotomy guides and customized osteosynthesis plates have been developed. These approaches allow direct transfer of the virtual plan to the surgical site and have been shown to improve surgical accuracy and predictability (6).

Mandibular reconstruction is commonly necessary after a traumatic injury, surgical resection of benign or malignant tumor, and osteomyelitis or osteoradionecrotic mandible (7). Although BSSO has long been performed and considered as a safe and reliable procedure in orthognathic surgery, its performance in a reconstructed mandible has been scarcely reported (8, 9). The altered anatomy, vascularity, and muscle attachments associated with reconstruction introduce additional technical and biological challenges that may affect surgical planning and execution.

This study reported a case of MMA using patient-specific osteotomy guides and osteosynthesis plates for a male OSA patient who previously received mandibular reconstruction with an osteocutaneous fibula flap.

## 2 Case presentation

### 2.1 Medical history

In 2022, a 53-year-old male patient was referred by an otorhinolaryngologist to the department of Oral and Maxillofacial Surgery for surgical consultation for OSA. Diagnosed with OSA in 2009, the patient, intolerant to continuous positive airway pressure (CPAP) therapy, initially chose mandibular advancement device (MAD) treatment. However, MAD became progressively less effective over the years. In 2017, the patient underwent the implantation of an upper airway stimulation system.

In 2018, the patient underwent segmental mandibulectomy (from left angulus mandibulae to right corpus mandibulae, targeting the region of the right canine) to address mandibular hemangioma. Reconstruction involved a free fibula osteocutaneous flap. After eight months, reconstruction plates were removed, and five implants were positioned for oral rehabilitation. In 2021, the patient presented to the Department of Otorhinolaryngology (ENT) and Head and Neck Surgery, seeking treatment for OSA after the mandibulectomy. The patient indicated a suboptimal response and intolerance to the upper airway stimulation system post-surgery, describing a sensation of excessive stimulation. At that time, his body mass index (BMI) was 30.8 kg/m^2^ and he scored 15 points in the Epworth Sleepiness Scale (ESS). An overnight polysomnography (PSG) test revealed a severe OSA [apnea hypopnea index (AHI) = 35.2 events/hour; Table 1]. After that, the ENT and Head and Neck surgeon referred the patient to the department of Oral and Maxillofacial Surgery to evaluate whether an MMA was possible, due to the previous mandibular reconstruction.

**Table 1:** Polysomnographic results before and after maxillomandibular advancement.

Variable	Baseline	Four-month follow-up
AHI, events/hour	35.2	17.6
AI, events/hour	4.1	9.9
HI, events/hour	31.1	7.7
AHI_supine,_ events/hour	45.1	22.8
AHI_non−supine,_ events/hour	20.5	8.7
Supine, % TST	59.7	63.4
AHIrem, events/hour	24	36.9
REM, % TST	15.3	14.2
ODI3%, events/hour	39.0	31.8
Mean SaO2, %	94	92
Min SaO2, %	81	71

AHI, apnea hypopnea index; AHI_supine_, apnea hypopnea index in supine postion; AHI_non−supine_, apnea hypopnea index in non-supine position; AI, apnea index; HI, hypopnea index; Mean SaO2, mean oxygen saturation; Min SaO2, minimum oxygen saturation; ODI, oxygen desaturation index; REM, rapid eye movement sleep; TST, total sleep time.

Upon intraoral exam at the department of Oral and Maxillofacial Surgery, the patient exhibited a partially edentulous mandible. The implant-supported restoration was not yet completed at that time. Based on the exam and the anamnesis, MMA using patient-specific osteotomy guides and osteosynthesis plates was suggested to the patient. The patient agreed to the treatment option with the intent of resolving his OSA.

### 2.2 Surgical planning

**Figure 1:**
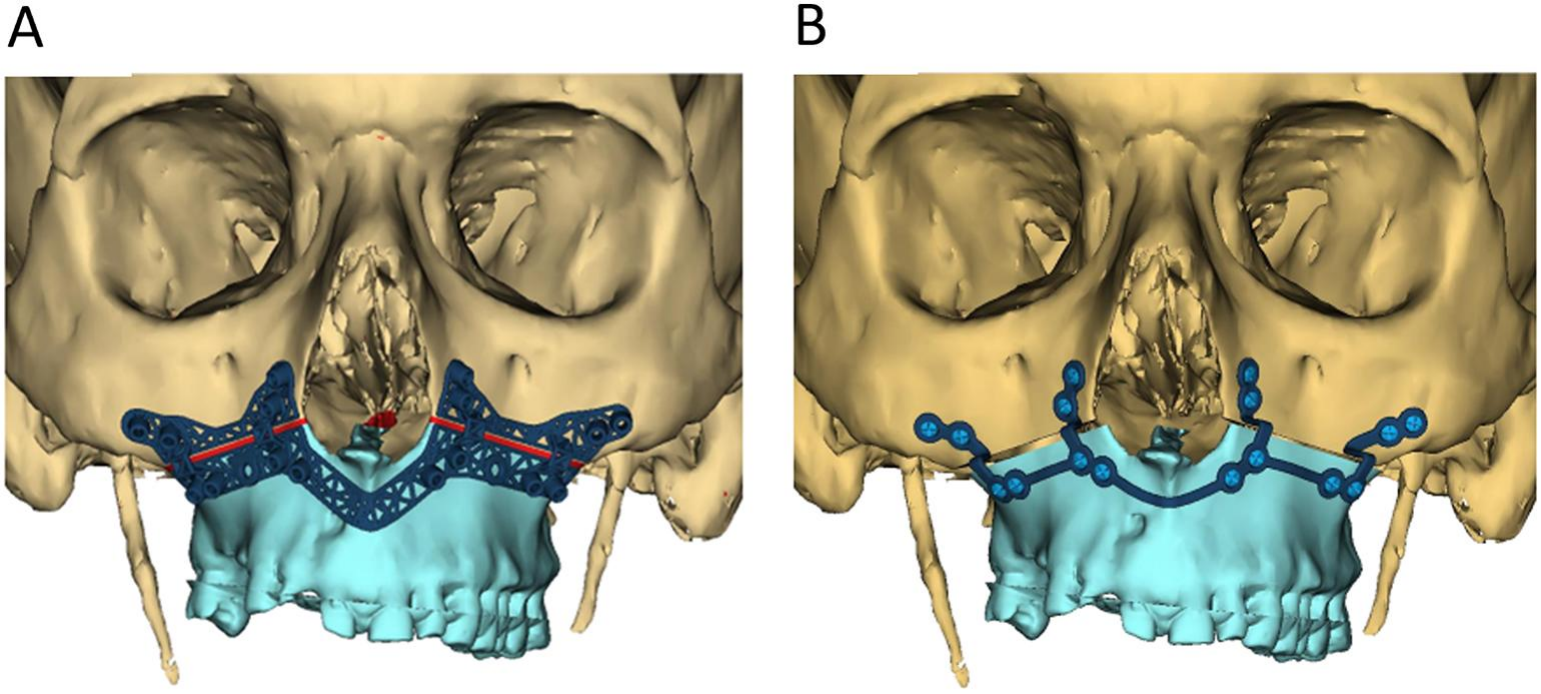
**(A)** Patient-specific titanium osteotomy guide for the maxilla; **(B)** titanium osteosynthesis plate for the maxilla.

**Figure 2:**
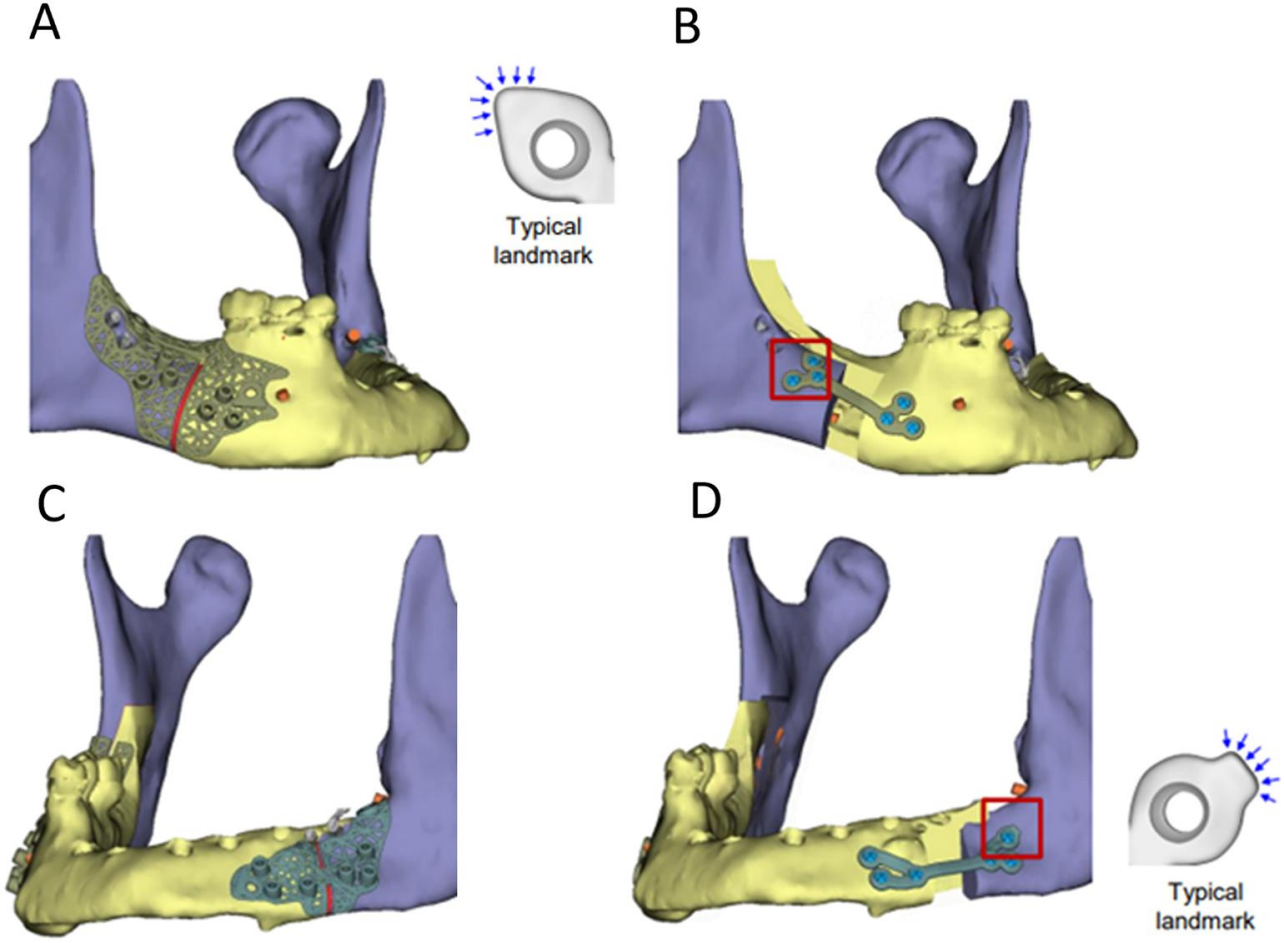
**(A)** Patient-specific titanium osteotomy guide for the mandible on the right side; **(B)** titanium osteosynthesis plate for the mandible on the right side; **(C)** patient-specific titanium osteotomy guide for the mandible on the left side (fibula segment); **(D)** titanium osteosynthesis plate for the mandible on the left side (fibula segment).

Virtual surgical planning (VSP) was performed by the attending surgeon and a clinical engineer. The maxilla and mandible were virtually osteotomized using Le Fort I and BSSO, respectively. The right sagittal split was designed based on the Hunsuck modification (10). The left sagittal split was adjusted to include a relatively short sagittal osteotomy of the fibula, stopping short of extending to the mandibular ramus. Osteotomy cutting guides were designed with embedded screw hole positions for osteosynthesis plates, strategically placed for easy access and sufficient bone fixation (Figures 1A, 2A,C). Planned movements of the maxilla and mandible were incorporated into the screw hole positions on the osteosynthesis plates (Figures 1B, 2B,D). The objective was to advance both the maxilla and mandible by 10 mm without altering the occlusion.

### 2.3 Maxillomandibular advancement

The patient underwent MMA surgery according to mandible-first protocol, 56 months after mandibular reconstruction. In the neomandible, the side-specific osteotomy guide was placed and temporarily fixed with two positioning screws. Afterwards, the horizontal, sagittal, and vertical cuts indicated by the guide were made with a piezosurgery device. After six fixation screw holes were pre-drilled, the guide was unscrewed. The split was completed with chisels in combination with sagittal splitters and separators. A BSSO on the right side was then performed following the osteotomy lines designed on the guide and six fixation screw holes were pre-drilled. Afterwards, the guide was removed and the split was finalized with chisels, sagittal splitters, and separators. After mobilization of the proximal and distal parts, both sides were fixed with the side-specific osteosynthesis plates and 12 bicortical screws in the pre-drilled screw holes.

**Figure 3:**
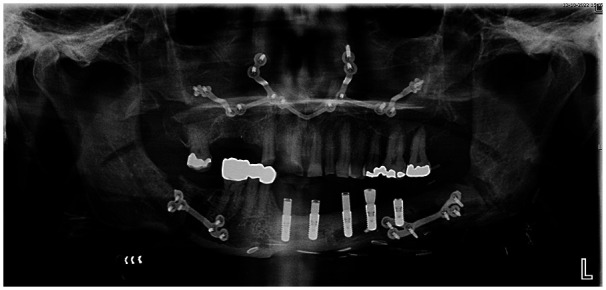
Panoramic radiograph two weeks after maxillomandibular advancement.

In the maxilla, the osteotomy guide was positioned in best fit and temporarily fixed with two positioning screws. A Le Fort I osteotomy cut indicated by the guide was made and 16 fixation screw holes were pre-drilled. Next, the guide was removed and the maxilla was down-fractured and mobilized with Rowe forceps. The osteosynthesis plate was used to reposition the maxilla, and to fix the maxilla in place with 16 monocortical screws (Figure 3).

The operation time was 210 min and estimated blood loss was 300 mL. The patient was admitted to post anesthesia care unit (PACU) for continuous monitoring in the first 24 h postoperatively and then was transferred to a general post-surgery ward for further recovery. The recovery was uneventful. The patient was discharged from the hospital three days after surgery.

### 2.4 Surgical results

At six-week follow-up, the patient reported improvement in sleep quality, almost without snoring and breathing pause after MMA. The postoperative BMI and ESS was 31.6 kg/m2 and three points, respectively. The four-month follow-up PSG showed a residual AHI of 17.6 events/hour. The summary of PSG results is shown in Table 1. Positional therapy was prescribed by an otorhinolaryngologist to further treat the patient's predominantly supine residual sleep apnea.

The patient also reported transient paraesthesia on the right side of the lower lip and in the infraorbital region postoperatively. Eight months after MMA, the patient underwent surgery under general anesthesia for removal of osteosynthesis material in the maxilla and in the right side of the mandible, due to recurrent epistaxis and maxillary sinus pressure, and loose screws, respectively. At the last follow-up (ten months after MMA), the patient was in a favorable state of recovery. He hoped to receive dental prosthesis as soon as possible. Regular follow-up appointments were arranged to monitor the patient's both long-term skeletal stability and respiratory outcomes.

## 3 Discussion

Orthognathic procedures on reconstruction sites have been reported to improve occlusal and aesthetic outcomes in patients who have undergone maxillomandibular reconstruction with bone flaps (8, 9, 11, 12). To the best of the authors’ knowledge, this is the first reported case wherein a patient who had undergone mandibular reconstruction with a free fibula flap, received MMA surgery for treating OSA.

To date, several orthognathic surgical techniques have been proposed for previously reconstructed mandibles with fibula flaps. In a study by Chang et al, vertical osteotomy was performed at the junction of the fibula and native mandible to reposition the grafted fibula, thus correcting malocclusion following reconstruction (11). In 2010 Gennaro and coworkers proposed the approach of step osteotomy on the fibula segment for correcting sagittal-length or vertical-height defect following reconstruction (12). In a case report by Kim et al, sagittal split osteotomy was performed on a previously reconstructed mandible with fibula flap (8), as the procedure used in the present case. Compared to vertical or step osteotomy, this procedure could allow a more sufficient bone interface to perform the entire range surgical movement, fast bone healing, and improve postsurgical stability.

For the patients who have undergone maxillomandibular reconstruction with bone flaps, a main difference with traditional orthognathic surgical patients is the possibility of absent teeth, which makes orthognathic surgery more challenging and less predictable in repositioning the maxilla and mandible. In this case, because the patient presented with multiple missing teeth, it was impossible to accurately perform regular surgical maneuvers such as the use of surgical splints and intermaxillary fixation. Therefore, a splintless orthognathic surgery technique using customized osteotomy guides and osteosynthesis plates was performed for the patient, which has shown to be accurate and predictable in edentulous patients in a previous study (13). In addition, when the patient presented to our clinic for managing OSA with MMA surgery, dental implants have been placed in the reconstructive mandible for oral rehabilitation. To ensure normal occlusal relationship in subsequent dental restoration, in surgical planning of this case, postoperative occlusal relationship was maintained in the same position as preoperative occlusion.

Various complications (e.g., bleeding, infection, and non-union) are associated with orthognathic surgery. For this patient, hardware removal, one the major complications after orthognathic surgery, was required in the maxilla due to recurrent epistaxis and sinus pressure and in the right mandible due to loose screws following MMA. Although some MMA patients may require secondary nasal surgery to address persistent nasal or sinus issues, in this case the epistaxis and sinus pressure resolved completely after hardware removal, without the need for additional nasal procedures, supporting an association between the local hardware irritation and the nasal symptoms. Additionally, the patient experienced transient neurosensory disturbances of the lower lip on the right side and the infraorbital region. Notably, no complications on fibula segment after BSSO were reported. This suggests that MMA can be considered a surgically safe treatment for OSA in selected patients with a previously reconstructed mandible when carefully planned and executed.

Upper airway stimulation had been previously implemented in this patient but became poorly tolerated following segmental mandibulectomy and fibula free flap reconstruction. This intolerance may be related to altered neuromuscular and biomechanical conditions of the tongue base and suprahyoid musculature following mandibular resection and reconstruction. This observation suggests that major mandibular surgery may influence effectiveness of hypoglossal nerve stimulation in OSA treatment and should be considered during preoperative counseling and multidisciplinary planning when determining the optimal sequencing of therapies.

In the present case, MMA resulted in a decrease of AHI from 35.2 to 17.6, which is regarded as successful for OSA treatment according to Sher's criteria (AHI drops at least 50% and is reduced below 20 postoperatively) (14). Besides, the ESS decreased from 15 to 3 points after MMA, demonstrating a favorable subjective outcome. However, it is also worth mentioning that for yet unknown reasons, postoperative PSG showed more apneic events accompanied by more desaturation compared with preoperative PSG, which necessitated additional therapy for OSA. Although three-dimensional airway volumetric analysis could have provided structural insight into the observed PSG changes and potential residual airway constriction, standardized pre- and postoperative airway segmentation was not available in this case. Given the residual apneic events occurred predominantly in supine position, positional therapy aiming to prevent patients from lying in the supine position was prescribed (15). This case suggests that each patient with OSA is unique and may have various response to treatment, and combined therapies for OSA can be indicated when necessary to deliver the best results for patients.

Finally, because altered vascularity and muscle attachments may predispose reconstructed mandibles to different relapse patterns compared with native bone (16), continued long-term monitoring is necessary to assess skeletal stability in the present case. Additionally, a follow-up PSG at 12–24 months is recommended to evaluate the durability of airway improvement and ensure that oxygen desaturation does not worsen over time.

## 4 Conclusion

In conclusion, this case report shows that MMA surgery using patient-specific osteotomy guides and osteosynthesis plates can be an effective and surgically safe treatment for severe OSA in patients with a previously reconstructed mandible.

## Data Availability

The original contributions presented in the study are included in the article/Supplementary Material, further inquiries can be directed to the corresponding author.
